# The prevalence and incidence of young onset dementia in the Netherlands: a prospective study in two catchment areas

**DOI:** 10.1186/s13195-026-02120-6

**Published:** 2026-06-22

**Authors:** Hanna E. Bodde, Jackie M. Poos, Guillaume H. P. R. Kolman, Angeline Bosman, Sten P. Willemsen, Kirsten Peetoom, Yolande A. L. Pijnenburg, Raymond Koopmans, Frans R. J. Verhey, Marjolein E. de Vugt, Christian Bakker, the PRECODE Study Group, Janne M. Papma

**Affiliations:** 1 Department of Neurology, Erasmus MC University Medical Center, Rotterdam, The Netherlands; 2 Department of General Practice, Erasmus MC University Medical Center, Rotterdam, The Netherlands; 3 Department of Epidemiology, Erasmus MC University Medical Center, Rotterdam, The Netherlands; 4 Department of Educational Development & Research, School of Health Professions Education, Faculty of Health, Medicine and Life Sciences, Maastricht University, Maastricht, The Netherlands; 5 Department of Neurology, Alzheimer Center Amsterdam, Vrije Universiteit Amsterdam, Amsterdam UMC, Amsterdam, The Netherlands; 6 Amsterdam Neuroscience, Neurodegeneration, Vrije Universiteit Amsterdam, Amsterdam UMC, Amsterdam, The Netherlands; 7 Department of Primary and Community Care, Radboudumc Alzheimer Center, Radboud University Medical Center, Nijmegen, the Netherlands; 8 Center for Specialized Geriatric Care, Joachim en Anna, Nijmegen, The Netherlands; 9 Department of Psychiatry and Neuropsychology, School for Mental Health and Neuroscience, Alzheimer Centre Limburg, Maastricht University, Maastricht, The Netherlands; 10 Groenhuysen, Center for Specialized Geriatric Care, Roosendaal, The Netherlands; 11 Department of Internal Medicine, Erasmus MC University Medical Center, Rotterdam, The Netherlands

## Abstract

**Background:**

Young-onset dementia (YOD), defined as dementia with symptom onset before the age of 65, is associated with distinct and often complex care needs that differ from those with late-onset dementia. Current national data on YOD incidence, prevalence, and demographic characteristics are limited, underscoring the importance of prospective studies to inform policy and tailored care services.

**Methods:**

We conducted two prospective one-year prevalence and incidence studies using active case-finding in two Dutch catchment areas, one urban and one more rural region, between 2020 and 2022. Individuals with YOD making use of care services were registered in an online database. Data on dementia subtype, living situation, and migration background were collected to explore regional variation.

**Results:**

We identified 372 individuals with YOD in the urban Rotterdam area, and 209 in the more rural Eindhoven-De Kempen area. Age-standardized prevalence per 100,000 individuals, standardized to the Dutch population, was 77.3 in the Rotterdam region, and 66.1 in the Eindhoven-De Kempen region for ages 30–69 years, and 46.3 and 36,9, respectively, for ages 30–64 years. Corresponding age-standardized incidence rates were 24.2 and 23.5 for ages 30–69 years, and 13.9 in both regions for ages 30–64 years. Alzheimer’s disease was the most common diagnosis in both areas. Compared with Eindhoven-De Kempen, the Rotterdam region had significantly more vascular dementia cases (*p* < 0.05 for incidence, and *p* < 0.001 for prevalence) and longer diagnostic delays (2.2 vs. 1.3 years between first GP consultation and diagnosis). Most individuals lived at home (76.4% and 85.4% resp. in Rotterdam and Eindhoven-De Kempen) and 30.8% in Rotterdam and 12.5% in Eindhoven-De Kempen had a migration background. Extrapolation of incidence figures suggests that between 14,655 and 20,340 individuals were living with YOD in the Netherlands in 2021.

**Conclusions:**

This study estimates the number of people living with YOD in the Netherlands. In addition, our findings show regional variation in dementia subtypes, a high proportion of people with YOD living at home, and a substantial proportion of people with YOD with a migration background. These insights underline the importance of region‑specific, person‑centered, culture sensitive YOD care at home.

**Supplementary Information:**

The online version contains supplementary material available at https://doi.org/10.1186/s13195-026-02120-6.

## Introduction

Dementia commonly affects older adults, but it can also develop at a younger age. This is referred to as young-onset dementia (YOD), defined as a symptom onset < 65 years [60]. YOD differs from dementia at older age in etiology [37, 38], symptom profile [5, 22], disease progression [22, 55], and its substantial impact on daily life [34]. As YOD affects people during an active life stage, it has extensive impact on employment, finances, and family responsibilities. To provide person-centered care for people with YOD, dementia healthcare services must be tailored to their specific needs. In the Netherlands, while specialized care for people with YOD is available, it remains unclear whether the capacity is aligned with the actual number and characteristics of people living with YOD [6, 40]. Achieving this will require robust national data on the incidence and prevalence of YOD, as well as a clearer overview of the characteristics of the affected population.

Internationally, several studies have researched the prevalence and incidence of YOD across different countries. Two comprehensive meta-analyses by Hendriks et al. synthesized the findings from 95 prevalence and 61 incidence studies, reporting age-standardized global prevalence and incidence estimates of respectively 119.0 per 100,000 population, and 11 per 100,000 person-years, for individuals aged 30 to 64 years [24, 26]. Based on these estimates, approximately 14,000—17,000 people in the Netherlands are currently living with YOD. However, findings across studies vary considerably, likely due to methodological differences. A key distinction is the use of retrospective registry-based data, large datasets from primary, secondary, or tertiary care, versus prospective case-finding methods, i.e. distributing questionnaires to healthcare facilities, engaging healthcare professionals, or working with key community informants. Both approaches have advantages and limitations: registry databases are prone to misclassification and incompleteness, whereas active case-finding is more accurate but resource-intensive and limited in scale. The methodological differences complicate efforts to draw general conclusions about regional YOD prevalence and incidence, and highlight the need for studies tailored to specific national or regional contexts, as existing international estimates may not reflect the national situation or capture country or region-specific characteristics of people living with YOD.

The present study aimed to (1) estimate the number of people living with YOD in the Netherlands, and (2) investigate differences in YOD prevalence and incidence—reflecting both existing and newly identified cases during a one-year period—between a highly urban region and a more rural region, together representing the Dutch population. In addition, we examined the characteristics of individuals with YOD, including their dementia subtype, living situation and country of origin. The findings are intended to support future care planning and policy development tailored to the needs of people with YOD in the Netherlands.

## Methods

### Study design

In this study we applied a prospective active case-finding approach to determine the one-year prevalence and incidence of YOD within two catchment areas in the Netherlands. We investigated differences in YOD prevalence and incidence in an urban region (the Rotterdam area) and a more rural area (the Eindhoven-De Kempen area), together reflecting the demographic diversity in the Netherlands. Within both areas, we contacted healthcare services to recruit healthcare professionals involved in the case-finding, diagnosis, care, and support of people with YOD. These healthcare professionals included dementia case managers, general practitioners (GPs), memory clinic staff such as neurologists, psychologists, and nurses, and healthcare professionals working in mental health care facilities, day care or nursing homes. During the course of one year, we asked them to register each person with YOD they encountered in an online registration database (Castor EDC, Amsterdam). In addition, we made use of regional hospital and case manager registries to identify people with a YOD diagnosis.

This study is part of the larger PRECODE (Prevalence Recognition and Care Pathways in Young Onset Dementia) project, which was initiated in 2018 to investigate the definition of YOD, the prevalence of people living with YOD in the Netherlands, their characteristics, and subsequently improve Dutch care pathways for people with YOD [24–27, 60, 61].

### Catchment areas

In determining the catchment areas, we considered both demographic factors, such as population size, population density, age distribution, migration background, and the presence of a YOD care network, to ensure access to relevant healthcare professionals and support services.

The first catchment area, referred to as the Rotterdam region, consists of the city of Rotterdam, the second-largest city in the Netherlands, and surroundings (see Supplementary Methods). This region is highly urbanized, with an average population density of 3154 inhabitants per km^2^, and a large proportion of people with a migration background (46,3%; Supplementary Table 1; Supplementary Fig. 2).

The second catchment area represents a more rural area and includes the city of Eindhoven along with the surrounding municipalities. We will refer to this region as the Eindhoven-De Kempen region. Including the city of Eindhoven in this catchment area was essential, as memory clinics and specialized residential care are located there. The Eindhoven-De Kempen region is highly representative of the Dutch population as a whole, with a migration background of 25% (compared with 23.1% nationwide) and an average population density of 591 inhabitants per km^2^, closely aligning with the national average of 519 inhabitants per km^2^. A more detailed description of both catchment areas is provided in the Supplementary Methods.

### Case-finding

Case-finding took place from February 1, 2020 to January 31, 2021 for the Rotterdam region and May 1, 2021 to April 30, 2022 for the Eindhoven-De Kempen region. We included individuals who received a dementia diagnosis before the age of 70, lived within the catchment area, and either received care during the measurement year or were placed on a care waiting list. The age cut-off of a diagnosis at 70 was chosen based on the definition of YOD of a symptom onset before the age of 65 [60], and taking into account a diagnostic delay of 4.4 years [62]. Individuals were excluded if their dementia was caused by Down syndrome, underlying infections such as HIV, alcohol-related conditions such as Wernicke’s encephalopathy or Korsakoff syndrome, Huntington’s disease, or traumatic brain injury, as these conditions follow different care pathways with designated healthcare facilities in the Netherlands.

The collected data included the clinical diagnosis, sex, age, age at diagnosis, date of first GP visit, date of diagnosis, living situation, and country of origin of individuals with YOD. Digits of the postal code were collected to verify whether the registered individual resided within the designated catchment area. Additionally, we gathered information about the healthcare professional who established the diagnosis, and the professional who registered the case, including their affiliated organization and registration date. In the Netherlands, YOD diagnoses are established at the memory clinic according to the Dutch multidisciplinary guideline for dementia diagnostics [14], which is based on multiple internationally accepted diagnostic criteria, i.e. McKhann [36] criteria for Alzheimer’s disease (AD), Rascovsky et al. [49] & Gorno-Tempini et al. [19] for frontotemporal dementia (FTD), and McKeith et al. [35] for Lewy Body Dementia [20]. We did not have access to the original clinical or diagnostic information used to establish the diagnosis at the memory clinic; instead, we relied on diagnoses and subtype classifications as recorded by the healthcare professionals involved. During registration, healthcare professionals selected the clinical diagnosis from a predefined drop-down menu including the most common dementia subtypes (i.e. AD, vascular dementia; VaD, behavioral variant FTD; bvFTD, Lewy Body Dementia; Primary progressive aphasia; PPA), or could select ‘other’ for rarer clinical diagnoses after which the diagnosis could be specified in free text.

### Rijnmond Primary Care Database 

To complement and validate our findings, we examined the prevalence and incidence of YOD within an available GP database in the Rotterdam region, the Rijnmond Primary Care Database (RPCD), which is part of the Integrated Primary Care Information project (IPCI; [12]). A detailed description of the database search and case identification procedure, including International Classification of Primary Care codes used, is provided in the Supplementary Methods.

### Statistical analysis

Crude yearly prevalence proportions and incidence rates were calculated using, respectively, the total number of identified cases, and the number of newly diagnosed cases within the measurement year as the numerator, and the corresponding population sizes as the denominator, stratified by 5-year age bands per 100,000. Next, crude and age-standardized prevalence and incidence estimates were calculated for three age groups: 30–64, 45–64, and 30–69 years. The 30–69 age range was chosen to align with the definition of YOD [60], while the 30–64 and 45–64 age ranges were selected to facilitate comparison with international studies. Prevalence and incidence estimates were age-standardized to the Dutch population in 2021, to the European Standard Population (ESP; [43]), and the World Standard Population (WSP; [1]) using direct standardization: Age-specific prevalence proportion and incidence rates were calculated for each age group, which were then weighted according to the age distribution of the standard population (Dutch population 2021, ESP or WSP). Age-standardized prevalence and incidence estimates for different age ranges were derived by combining the weighted rates across age groups.

Individuals were included in the calculations if their age fell within the specified range on a predefined census date, set at the midpoint of the measurement year. For individuals registered in the Rotterdam region, this date was August 1, 2020; for individuals registered in the Eindhoven-De Kempen region, November 1, 2021, and for individuals in the RPCD, July 1, 2020. We calculated prevalence proportions and incidence rates for the total group (all diagnoses), and separately for the most prevalent dementia subtypes (AD, VaD and FTD). For FTD, both bvFTD diagnoses and PPA were included. Statistical analyses were conducted using R Statistical Software (v4.3.0; [47]). We assessed differences in age-standardized prevalence proportions and incidence rates between the Rotterdam and Eindhoven-De Kempen areas by comparing the proportions and rates using a normal approximation. We assumed normally distributed standardized proportions and rates, and calculated corresponding 95% confidence intervals. To examine differences in demographic and clinical characteristics between individuals registered in the two regions, we used Shapiro–Wilk tests to assess the distribution of continuous variables (e.g., age at diagnosis, age at census date, socioeconomic status). Socioeconomic status (SES) was derived from the Dutch Central Bureau for Statistics [7], which calculates neighborhood‑level SES scores based on the financial well‑being, educational level, and recent employment history of private households within specific postal‑code areas. As these variables deviated significantly from normality, non-parametric Mann–Whitney U tests were used. For categorical variables (e.g., migration background, sex, living situation, diagnosing professional), chi-square tests were conducted. Living situation was dichotomized into ‘living at home’ versus ‘living in institutional care facility’. An alpha level of 0.05 was used to determine statistical significance, and effect sizes were calculated where relevant.

To estimate the number of people living with YOD in the Netherlands, we applied two approaches:

- *Approach 1—Extrapolation of prevalence proportions:* Age-standardized prevalence proportions for individuals aged 30–69 years were extrapolated to the national level by multiplying regional estimates by the total Dutch population within the same age range.
- *Approach 2—Estimating the national YOD prevalence combining incidence and disease duration:* We combined incidence rates for individuals aged 30 −69 years with estimates of median disease duration for YOD to derive a point prevalence. Estimates of disease duration were based on three recent Dutch studies [17, 28, 51], which reported median disease durations ranging from 6.9 years to 9.3 years.

For both approaches, we used age-standardized outcomes to correct for differences in age distribution between the study regions and the total Dutch population. Applying these methods to data from the Rotterdam and Eindhoven-De Kempen areas resulted in a national prevalence range, with minimum and maximum estimates reflecting the regional variation observed in the underlying data.

## Results

### Case-finding

Within the Rotterdam area, 567 case registrations were made within a 1-year time period. Of these, 116 registrations were duplicates. Furthermore, cases were excluded based on registered diagnoses meeting the exclusion criteria (*n* = 11), missing of essential information (*n* = 4), living outside of the catchment area (*n* = 39), being older than 70 at the time of diagnosis (*n* = 17), and for being diagnosed after the year-measurement period (*n* = 8), resulting in *n* = 372 unique cases. Registrations were made by dementia case managers (13.2%), healthcare professionals working in memory clinics (33.8%), mental healthcare facilities (24.5%), daycare facilities (14.7%), nursing homes (13.2%), and GPs (0.5%).

In the Eindhoven-De Kempen area, 309 registrations were recorded. Sixty-seven of these registrations were duplicates. Registrations were excluded due to a diagnosis not meeting the exclusion criteria (*n* = 4), the missing of essential information (*n* = 3), age over 70 at the time of diagnosis (*n* = 12), residence outside the catchment area (*n* = 14), resulting in *n* = 209 unique cases. Registrations were made by dementia case managers (46.7%, including 3.7% from a case manager registry), memory clinics (29.9%), daycare facilities (13.3%), nursing homes (8%), and GPs (1.9%).

### Study population characteristics

**Fig. 1:**
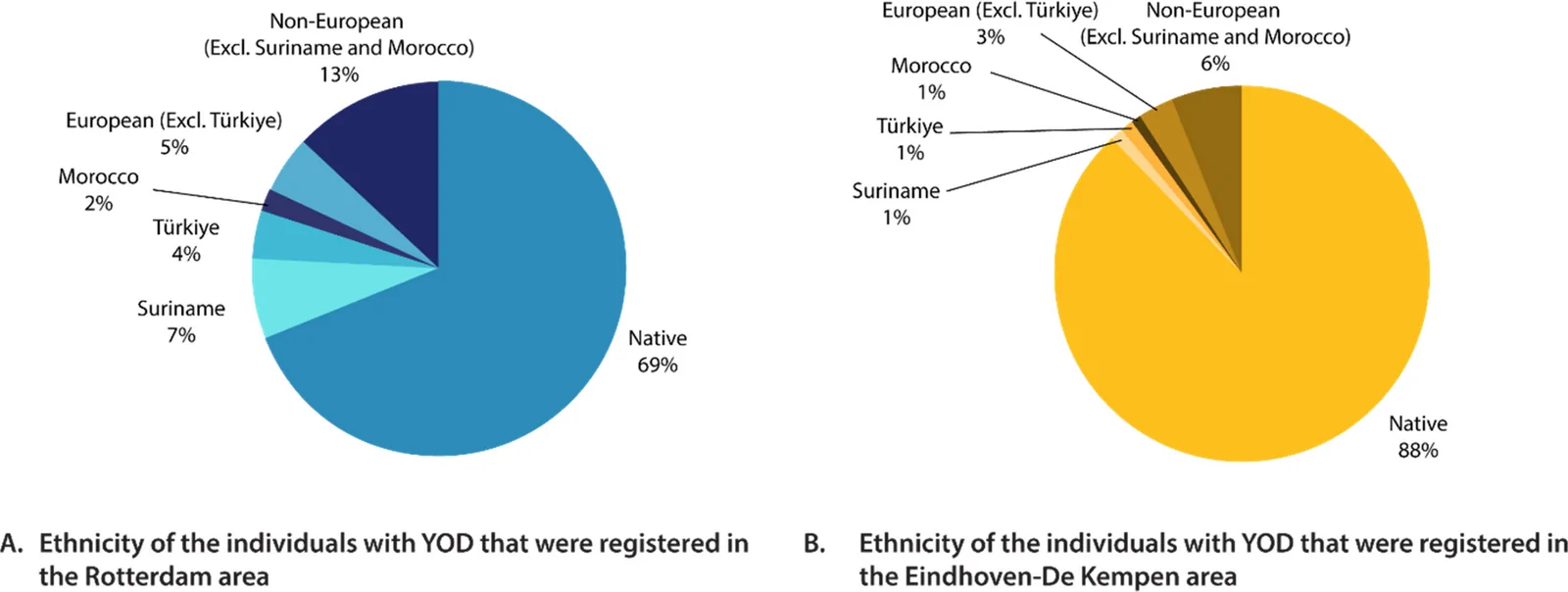
Country of origin of registered individuals with YOD and migration background. Data was missing for 64 individuals in the Rotterdam area, resulting in a sample size of 308. Data was missing for 49 individuals in the Eindhoven-De Kempen area, resulting in a sample size of 160

In the Rotterdam area, we identified 372 individuals with YOD (Table 1), of whom 100 were newly diagnosed during the measurement year. Within the Eindhoven-De Kempen area, a total of 209 individuals with YOD were identified (Table 1), of whom 68 were newly diagnosed during the measurement year. Within both catchment areas, the majority of individuals with YOD were living at home (85.4% in the Rotterdam area and 76.4% within the Eindhoven-De Kempen area). Notably, 30.8% of individuals with YOD in the Rotterdam area, and 12.5% in the Eindhoven–De Kempen area had a migration background, originating from a range of different countries (Fig. 1). The Rotterdam area included individuals in a younger age category (35–44 years), which was not represented in the Eindhoven–De Kempen area (Supplementary Table 2), and showed a lower minimum age at diagnosis (23 vs. 42 years; Table 1), although this difference was not statistically significant.

**Table 1:** Baseline characteristics of all included YOD persons in both catchment areas

	**Rotterdam area**	**Eindhoven-De Kempen area**
Identified persons with YOD	372	209
Age at diagnosis in years mean, SD	61.1 ± 7.8^1^	61.9 ± 5.9^2^
Age at diagnosis in years, range	23–69^1^	42–69^2^
Age at census date in years; mean, SD	62.9 ± 8.0	63.9 ± 5
Time between first GP visit and diagnosis in years; mean, SD	2.2 ± 2.5^3^	1.3 ± 1.7^4^
Time between first GP visit and diagnosis in years, range	0—18.5^3^	0—9.8^4^
Sex, Female	174 (46.9%)	114 (54.8%)^5^
People with a Dutch/native background	213 (69.2%)^6^	140 (87.5%)^7^
People with a migration background*	95 (30.8%)^6^	20 (12.5%)^7^
Social Economic Status (SES) score; mean, SD **	−0.25 ± 0.27	0.04 ± 0.21
Living situation		
*Independent, alone*	57 (18.9%)^8^	28 (17.7%)^9^
*Independent, with spouse and/or children*	173 (57.5%)^8^	95 (60.1%)^9^
*Independent, unknown*	-	12 (7.6%)^9^
*Nursing home*	69 (22.9%)^8^	20 (12.7%)^9^
*Seniorflat*	2 (0.7%)^8^	1 (0.6%)^9^
*Other*	-	2 (1.3%)^9^
Diagnosis		
*Alzheimer’s disease*	185 (52.3%)^10^	106 (54.1%)^11^
*Vascular dementia*	93 (26.3%)^10^	22 (11.2%)^11^
*Frontotemporal dementia*	30 (8.4%)^10^	28 (14.3%)^11^
*Behavioral variant FTD*	26 (7.3%)^10^	17 (8.7%)^11^
*PPA (incl. semantic dementia/nfvPPA)*	4 (1.1%)^10^	11 (5.6%)^11^
*Lewy Body dementia*	10 (2.8%)^10^	4 (2.0%)^11^
*Mixed dementia*	9 (2.5%)^10^	6 (3.1%)^11^
*Parkinson’s disease dementia*	7 (2.0%)^10^	13 (6.6%)^11^
*Other diagnosis*^*a*^	4 (1.1%)^10^	8 (4.1%)^11^
*Unknown diagnosis*	16 (4.5%)^10^	9 (4.6%)^11^
Diagnosed by		
*Neurologist*	265 (71.8%)^12^	159 (85.9%)^13^
*Geriatrician*	22 (6.0%)^12^	15 (8.1%)^13^
*Psychiatrist*	44 (11.9%)^12^	3 (1.6%)^13^
*Psychologist*	14 (3.8%)^12^	2 (1.1%)^13^
*Specialist geriatric medicine*	22 (6.0%)^12^	2 (1.1%)^13^
*General practitioner*	2 (0.5)^12^	4 (2.2%)^13^

*Abbreviations:* *YOD* young-onset dementia, *y* years, *SD* standard deviation, *FTD* frontotemporal dementia, *PPA* primary progressive aphasia, *nfvPPA* non fluent variant of PPA

^1^Data was missing for 4 individuals, resulting in a sample size of 368

^2﻿^Data was missing for 15 individuals, resulting in a sample size of 194

^3﻿^Data was missing for 75 individuals, resulting in a sample size of 297

^4﻿^Data was missing for 104 individuals, resulting in a sample size of 105

^5﻿^Data was missing for 1 individuals, resulting in a sample size of 208

^6﻿^Data was missing for 64 individuals, resulting in a sample size of 308

^7﻿^Data was missing for 49 individuals, resulting in a sample size of 160

^8﻿^Data was missing for 71 individuals, resulting in a sample size of 301

^9﻿^Data was missing for 51 individuals, resulting in a sample size of 158

^10^Data was missing for 18 individuals, resulting in a sample size of 354

^11^Data was missing for 13 individuals, resulting in a sample size of 196

^12^Data was missing for 3 individuals, resulting in a sample size of 369

^13^Data was missing for 24 individuals, resulting in a sample size of 185

^a^Other diagnoses were dementia due to multiple sclerosis (MS; *n *= 3), multiple system atrophy (MSA; *n* = 2), Huntington’s disease (*n* = 2), corticobasal degeneration (CBD; *n* = 2), posterior cortical atrophy (PCA; *n* = 1), cerebral amyloid angiopathy (CAA; *n* = 1), and progressive supranuclear palsy (PSP; *n* = 1)

^*^See Fig. 1

^**^This score was calculated by the Central Bureau for Statistics (CBS) in the Netherlands and is based on the financial well-being, educational level and recent work history of private households in certainpostal codes

While age at census date and sex were comparable between the two catchment areas (Table 1), several socio‑demographic and care‑related characteristics differed. The Rotterdam area showed a higher proportion of individuals with a migration background (*p* < 0.001), a longer interval between first GP visit and diagnosis (*p* < 0.001), a greater proportion of individuals living in institutional care (*p* = 0.023), lower SES (*p* < 0.001, r = 0.49), and a different distribution of diagnosing professionals (*p* < 0.001) compared with the Eindhoven–De Kempen area.

### Prevalence and incidence of YOD

**Fig. 2:**
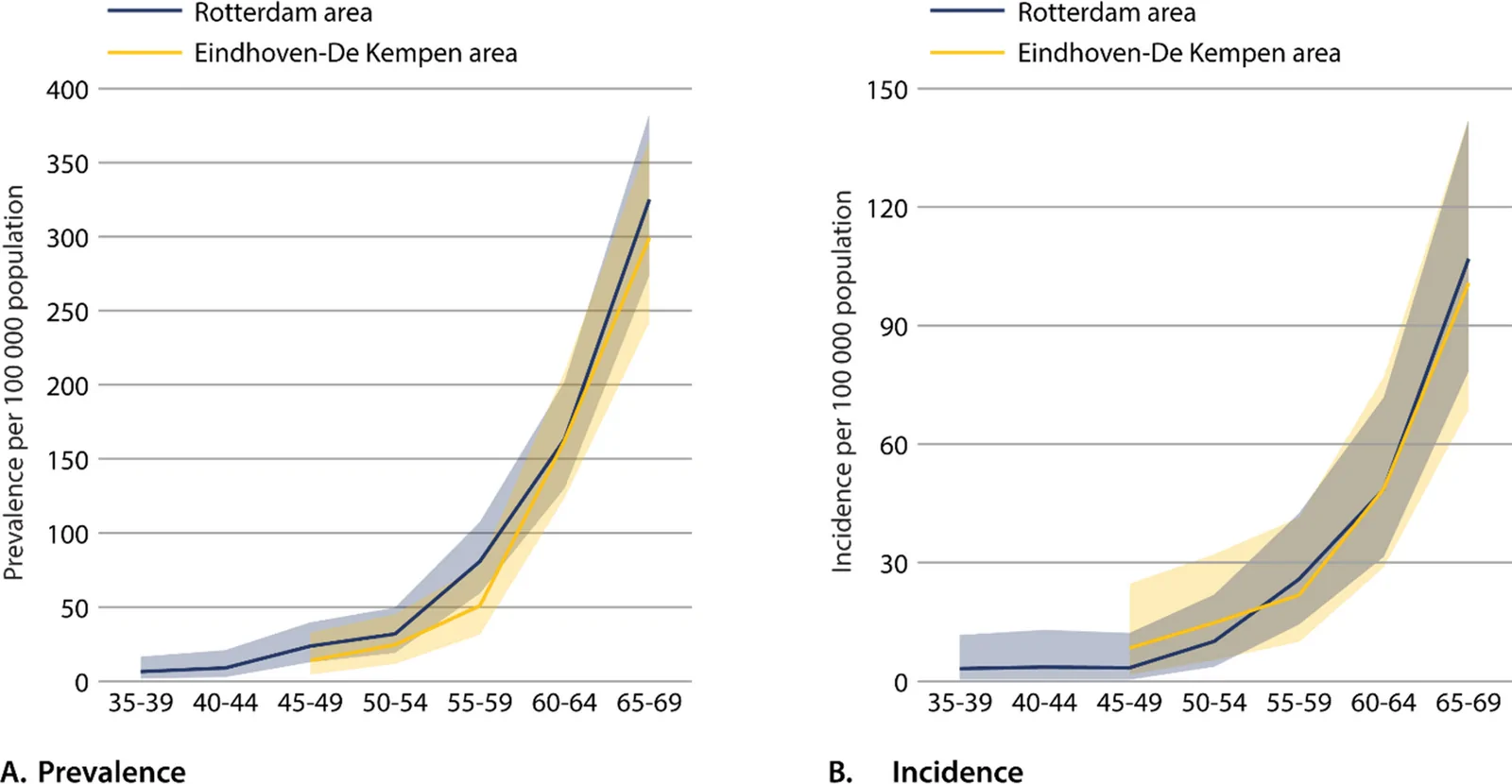
1-year crude prevalence and incidence per 100 000 individuals. **A** Prevalence in Rotterdam area and Eindhoven-De Kempen area. **B** Incidence in Rotterdam area and Eindhoven-De Kempen area

Crude and age-standardized prevalence and incidence of YOD increased with age (Fig. 2, Supplementary Table 2). Age‑standardized incidence rates (standardized to the Dutch population) were highly comparable between the two regions (24.2 per 100,000 in the Rotterdam area versus 23.5 per 100,000 in the Eindhoven-De Kempen area for ages 30–69 years; Table 2). Age‑standardized prevalence was higher in the Rotterdam area than in the Eindhoven-De Kempen area (77.3 vs. 66.1 per 100,000 for ages 30–69 years). However, neither age‑standardized incidence nor prevalence differed significantly between the two regions across any of the examined age groups (30–64, 45–64, and 30–69 years). Age-standardization to the ESP and WSP resulted in lower prevalence and incidence estimates than standardization to the Dutch population (Table 2).

**Table 2:** Crude and age-standardized prevalence and incidence per 100,000 individuals

Age range	Prevalence				Incidence			
	**Crude**	**NL**^a^	**ESP**^a^	**WSP**^a^	**Crude**	**NL**^a^	**ESP**^a^	**WSP**^a^
Rotterdam								
30–64	41.5 (35.6–48.2)	46.3 (39.6–53.7)	42.6 (36.5–49.4)	34.0 (29.1–39.5)	12.5 (9.3–16.4)	13.9 (10.4–18.3)	12.8 (9.6–16.8)	10.2 (7.6–13.5)
45–64	71.8 (61.2–83.7)	73.8 (62.9–86.0)	71.5 (60.9–83.3)	65.5 (55.8–76.4)	21.0 (15.5–27.9)	21.7 (16.0–28.8)	20.9 (15.4–27.7)	18.9 (13.9–25.1)
30–69	68.6 (61.3–76.6)	77.3 (69.0–86.3)	72.2 (64.5–80.6)	53.6 (47.8–59.9)	21.5 (17.5–26.2)	24.2 (19.7–29.5)	22.7 (18.4–27.6)	16.7 (13.6–20.4)
Eindhoven								
30–64	36.6 (29.6–44.7)	36.9 (29.9–45.1)	33.5 (27.2–41.0)	25.7 (20.7–31.4)	13.7 (9.6–19.0)	13.9 (9.7–19.2)	12.7 (8.9–17.6)	10.0 (7.0–13.9)
45–64	62.2 (50.3–75.9)	61.6 (49.9–75.2)	59.5 (48.2–72.6)	53.5 (43.2–65.5)	23.3 (16.3–32.3)	23.1 (16.2–32.0)	22.5 (15.8–31.2)	20.9 (14.5–29.0)
30–69	65.0 (56.1–74.8)	66.1 (57.0–76.1)	61.4 (53.0–70.7)	44.1 (38.0–50.9)	23.1 (18.0–29.3)	23.5 (18.3–29.8)	21.9 (17.0–27.8)	16.1 (12.5–20.5)

^a^Age-standardized prevalence and incidence was calculated by means of direct standardizations against the Dutch population in 2021 (NL), the European Standard Population (ESP) and the World Standard Population (WSP)

### Diagnostic subtypes

Across both catchment areas and all examined age groups (30–64, 45–64, and 30–69 years), AD was the most prevalent dementia subtype (Table 3). In the Rotterdam area, VaD was the second most prevalent subtype, followed by FTD. In the Eindhoven-De Kempen area, FTD was the second most prevalent subtype, followed by VaD (Table 3). VaD was significantly less common in the Eindhoven-de Kempen area compared to the Rotterdam area for all age groups (Table 3; *p* < 0.05 for incidence, and *p* < 0.001 for prevalence). No significant regional differences were found in the Dutch age-standardized prevalence or incidence of AD or FTD.

**Table 3:** One year prevalence and incidence of the most common clinical diagnoses of YOD

Age range	Prevalence				Incidence			
	**Crude**	**NL** ^a, c^	**ESP** ^a^	**WSP** ^a^	**Crude**	**NL** ^a^	**ESP** ^a^	**WSP** ^a^
**Rotterdam area**								
**AD**^b^								
30–64	19.2 (15.2–23.9)	21.6 (17.1–26.8)	19.7 (15.6–24.5)	15.4 (12.2–19.2)	4.8 (2.9—7.4)	5.5 (3.3–8.4)	4.9 (3.0–7.6)	3.7 (2.3–5.8)
45–64	34.2 (27.0–42.6)	35.2 (27.8–43.9)	34.0 (26.9–42.4)	30.9 (24.4–38.6)	8.8 (5.3—13.5)	9.1 (5.6–14.1)	8.7 (5.3–13.5)	7.8 (4.7–12.0)
30–69	34.5 (29.4–40.3)	39.1 (33.3–45.7)	36.4 (31.0–42.6)	26.4 (22.5–30.9)	9.3 (6.8—12.6)	10.7 (7.7–14.4)	9.9 (7.2–13.3)	7.0 (5.1–9.4)
**VaD**^b^								
30–64	13.0 (9.7–16.9)	14.2*** (10.7–18.6)	13.3 (10.0–17.4)	11.1 (8.3–14.6)	4.6 (2.7—7.1)	5.0* (3.0–7.8)	4.7 (2.8–7.3)	4.0 (2.4–6.3)
45–64	21.0 (15.5–27.9)	21.5*** (15.9–28.5)	20.9 (15.4–27.8)	19.6 (14.4–26.0)	6.6 (3.7—10.8)	6.8* (3.8–11.2)	6.5 (3.6–10.7)	6.0 (3.3–9.9)
30–69	17.6 (14.0–21.9)	19.5*** (15.5–24.2)	18.4 (14.6–22.8)	14.5 (11.5–18.1)	7.0 (4.8—9.8)	7.7* (5.3–10.9)	7.3 (5.0–10.3)	5.7 (3.9–8.1)
**FTD**								
30–64	3.1 (1.7–5.3)	3.5 (1.8–5.9)	3.2 (1.7–5.5)	2.6 (1.4–4.5)	1.0 (0.3—2.5)	1.1 (0.3–2.8)	1.0 (0.3–2.5)	0.7 (0.2–1.8)
45–64	5.3 (2.7–9.2)	5.4 (2.8–9.4)	5.3 (2.7–9.2)	4.9 (2.5–8.5)	1.8 (0.5—4.5)	1.8 (0.5–4.7)	1.7 (0.5–4.5)	1.5 (0.4–3.8)
30–69	5.2 (3.3–7.8)	5.8 (3.7–8.7)	5.5 (3.5–8.2)	4.1 (2.6–6.2)	1.5 (0.6–3.1)	1.7 (0.7–3.6)	1.6 (0.6–3.3)	1.1 (0.5–2.3)
**Eindhoven-De Kempen area**								
**AD**^b^								
30–64	18.7 (13.8–24.7)	18.8 (13.9–24.9)	17.0 (12.6–22.5)	12.8 (9.5–16.9)	6.9 (4.1–10.9)	6.9 (4.1–10.9)	6.2 (3.7–9.8)	4.8 (2.8–7.6)
45–64	31.7 (23.5–41.9)	31.4 (23.2–41.5)	30.1 (22.3–39.8)	26.7 (19.7–35.3)	11.7 (6.9–18.4)	11.5 (6.8–18.2)	11.0 (6.5–17.4)	10.0 (5.9–15.8)
30–69	32.3 (26.1–39.5)	32.8 (26.6–40.1)	30.4 (24.6–37.1)	21.7 (17.5–26.5)	11.9 (8.3–16.6)	12.1 (8.4–16.8)	11.2 (7.8–15.6)	8.1 (5.6–11.3)
**VaD**^b^								
30–64	3.8 (1.8–7.0)	3.9*** (1.9–7.1)	3.5 (1.7–6.5)	2.8 (1.3–5.1)	1.1 (0.2–3.3)	1.2* (0.2–3.4)	1.1 (0.2–3.1)	0.8 (0.2–2.4)
45–64	6.5 (3.1–11.9)	6.4*** (3.1–11.8)	6.3 (3.0–11.5)	5.7 (2.7–10.6)	1.9 (0.4–5.7)	1.9* (0.4–5.7)	1.9 (0.4–5.5)	1.7 (0.3–5.0)
30–69	7.1 (4.4–10.9)	7.3*** (4.5–11.1)	6.8 (4.2–10.4)	4.9 (3.0–7.5)	2.4 (1.0–4.9)	2.4* (1.0–5.0)	2.3 (0.9–4.7)	1.6 (0.6–3.3)
**FTD**								
30–64	6.5 (3.8–10.4)	6.6 (3.8–10.5)	6.1 (3.5–9.7)	4.9 (2.8–7.9)	2.3 (0.8–5.0)	2.3 (0.9–5.1)	2.2 (0.8–4.9)	1.9 (0.7–4.3)
45–64	11.0 (6.4–17.6)	10.9 (6.4–17.5)	10.8 (6.3–17.2)	10.2 (5.8–16.4)	3.9 (1.4–8.5)	3.9 (1.4–8.5)	3.9 (1.4–8.6)	4.0 (1.4–8.9)
30–69	8.5 (5.5–12.6)	8.6 (5.6–12.7)	8.1 (5.2–11.9)	6.2 (4.0–9.3)	3.1 (1.4–5.8)	3.1 (1.4–5.9)	3.0 (1.4–5.7)	2.4 (1.1–4.7)

*Abbreviations: AD* Alzheimer’s disease, *VaD* vascular dementia, *FTD* frontotemporal dementia

^a^Age-standardized prevalence and incidence was calculated by means of direct standardizations against the Dutch population in 2021 (NL), the European Standard Population (ESP) and the World Standard Population (WSP)

^b^A total of 15 individuals with a mixed diagnosis (AD + VaD) were excluded from the analyses

^c^Prevalence and incidence age-standardized against the Dutch population were statistically compared between the Rotterdam and the Eindhoven-De Kempen catchment areas

^*^Statistically significant difference (*p* < 0.05) in age-adjusted incidence of VaD between the Rotterdam and Eindhoven-De Kempen areas

^***^Statistically significant difference (*p* < 0.05) in age-adjusted prevalence of VaD between the Rotterdam and Eindhoven-De Kempen areas

### Estimates of the prevalence of YOD in the Netherlands

The total population size of individuals aged 30 to 69 years living in the Netherlands reported by the Central Bureau of Statistics (CBS) for 2021, were 9,037,764 individuals. Based on this number, we applied the age-standardized prevalence figures from our study to the Dutch population. This resulted in an estimated range of 5974 to 6986 individuals with YOD in the Netherlands. When combining the incidence rates with the estimated disease durations of 6.9 to 9.3 years [17, 28, 51], this yielded an estimated prevalence range of 14,655 to 20,340 individuals with YOD in the Netherlands in 2021.

### Rijnmond Primary Care Database 

The search within the RPCD for the Rotterdam region yielded 1,595 records between 2010–2020, including 554 identified via ICPC code P70 (dementia), and 1,041 via ICPC P20 (memory, concentration or orientation problems) combined with keywords in free-text notes. After manual identification by HB, 381 cases were confirmed as YOD, representing 57% of the P70 records and 6% of the P20 plus keyword records. Of these, 89 were still registered in the database in 2020 and were aged 30–69 years at the census date.

The overall age-standardized prevalence calculated from the RCPD data were comparable to the prevalence figures in the Rotterdam area in all age groups (Table 4; Supplementary Fig. 3). Age-standardized incidence was 17.3 per 100,000 (95% CI: 11.0 to 26.5) in the 30–69 year group, which is somewhat lower than the Rotterdam area (24.2 per 100,000), whilst incidence rates for the other age groups (30–64 and 45–64) were comparable. In the database data, AD was the most prevalent cause of dementia, followed by VaD, and FTD (Table 4).

**Table 4:** One year prevalence and incidence of YOD and the most common etiologies of YOD in the Rijnmond Primary Care Database

	**Population**	**Personyears**	**Prevalence**			**Incidence**		
			**n**	**Crude prevalence (95% CI)**	**Age-standardized prevalence**^**a**^** (95% CI)**	**n**	**Crude incidence (95% CI)**	**Age-standardized incidence**^**a**^** (95% CI)**
Total								
30–64	99685	116,523	43	43.1 (31.2–58.1)	44.2 (32.0–59.6)	16	13.7 (7.9–22.3)	14.2 (8.1–23.0)
45–64	59478	69,401	43	72.3 (52.3–97.4)	73.7 (53.3–99.3)	16	23.1 (13.2–37.4)	23.6 (13.5–38.4)
30–69	113629	130,081	89	78.3 (62.9–96.4)	76.0 (61.0–93.5)	22	16.9 (10.6–25.6)	17.5 (11.0–26.5)
AD								
30–64	99685	116,523	19	19.1 (11.5–29.8)	19.6 (11.8–30.5)	8	6.9 (3.0–13.5)	7.1 (3.1–13.9)
45–64	59478	69,401	19	31.9 (19.2–49.9)	32.6 (19.6–50.9)	8	11.5 (5.0–22.7)	11.8 (5.1–23.2)
30–69	113629	130,081.9	39	34.3 (24.4–46.9)	33.3 (23.7–45.6)	10	7.7 (3.7–14.1)	8.0 (3.8–14.6)
VaD								
30–64	99685	116,523	7.0	7.0 (2.8–14.5)	7.2 (2.9–14.9)	5.0	4.3 (1.4–10.0)	4.4 (1.4–10.3)
45–64	59478	69,401	7.0	11.8 (4.7–24.3)	12.0 (4.8–24.8)	5.0	7.2 (2.3–16.8)	7.4 (2.4–17.2)
30–69	113629	130,081	16.0	14.1 (8.1–22.9)	13.6 (7.8–22.1)	5.0	3.8 (1.3–9.0)	3.9 (1.3–9.2)
FTD								
30–64	99685	116,523	4	4.0 (1.1–10.3)	4.1 (1.1–10.5)	1	0.9 (0.0-4.8)	0.9 (0.0-5.1)
45–64	59478	69,401	4	6.7 (1.8-17.2–12.2)	6.9 (1.9–17.6)	1	1.4 (0.0-8.0)	1.5 (0.0-8.4)
30–69	113629	130,081	8	7.4 (3.0–13.9)	6.9 (3.0–13.5)	1	0.8 (0.0-4.3)	0.8 (0.0-4.5)

*Abbreviations: AD* Alzheimer’s disease, *VaD* vascular dementia, *FTD* frontotemporal dementia

^a^Age-standardized prevalence and incidence was calculated by means of direct standardizations against the Dutch population in 2021

^b^A total of 10 individuals were with a mixed diagnosis (AD + VaD) were not included in the analyses for AD and VaD

## Discussion

We estimated the prevalence and incidence of YOD in the Netherlands using an active case-finding approach across two demographically distinct regions: the urban Rotterdam area and the more rural Eindhoven-De Kempen area. We identified a total of 581 individuals with YOD in both regions, of which 168 were newly diagnosed during the measurement year. Age-standardized prevalence to the Dutch population ranged from 36.9 (Eindhoven-De Kempen region) to 46.3 (Rotterdam region) per 100,000 individuals aged 30–64 years, and incidence was 13.9 per 100,000 both in the Rotterdam and the Eindhoven-De Kempen area. Based on the observed incidence and average disease duration, we estimated that between 14,655 and 20,340 individuals aged 30–69 were living with YOD in the Netherlands in 2021. Validation with RPCD data showed estimates that were largely consistent with those observed in the Rotterdam area. In both areas, AD was the most common diagnosis. In the Rotterdam area, this was followed by VaD and FTD, whereas in the Eindhoven-De Kempen area FTD was the second most common diagnosis.

The ESP and WSP age-standardized prevalence proportions for YOD found in our study were lower than those reported in international YOD prevalence studies, which ranged from 54.0 to 141.2 per 100,000 individuals aged 30–64 years [8, 23, 24, 29, 31, 54, 66]. Differences between studies should be interpreted in the context of diagnostic practice at the time, as changes in diagnostic criteria, diagnostic tests and societal dementia awareness over time may influence YOD prevalence and incidence estimates. Several methodological differences may further explain this discrepancy, like differences in inclusion and exclusion criteria or variations in data collection. Registry-based studies, such as Ryan et al. [54], depend on the accuracy and completeness of existing records, which can lead to under- or overestimation of prevalence proportions and incidence rates since this data is not specifically collected for research purposes. This limitation was evident in our use of the RPCD data, where manual verification revealed that only 57% of individuals with an ICPC code P70 (diagnosis of dementia) prior to age 70 were true YOD cases, highlighting the importance of manual verification in registry-based studies.

The influence of methodological choices is illustrated by comparison of our results to the European study by Logroscino et al. [33]. In this retrospective registry-based study, conducted in 2018–2019, the FTD incidence reported for the Rotterdam Rijnmond region, a region largely overlapping with our Rotterdam catchment area, was 0.87 per 100,000 in individuals aged 45–64 years, whereas our actively ascertained incidence estimates for the same age range was more than twice as high (1.8 per 100,000). This contrast highlights how differences in case-finding strategies potentially affect estimates, even across similar populations over a comparable time frame.

Other factors contributing to international variation in prevalence may include differences in diagnostic criteria [24], diagnostic delays [18, 46], age distribution within the population, and lack of age-standardized reporting [26]. In addition, variation in care pathways and referral practices across countries, including the management of conditions like vascular dementia within stroke services rather than dementia services, can play a role [3]. The limited observation period of our study may have also led to an underestimation of prevalence, as only individuals with YOD seen within a one-year window were included, potentially excluding those diagnosed earlier though not yet receiving formal care after diagnosis.

The ESP and WSP age-standardized incidence rates in our study are slightly lower than international figures, ranging from 13.2–17.8 per 100,000 individuals aged 30–64 [8, 15, 16, 29, 32]. Incidence figures derived from our study may provide a more reliable basis for estimating the total number of people living with YOD than prevalence figures specifically for the Netherlands. First, unlike prevalence, incidence is not influenced by the duration of the study period. Second, all memory clinics in the study regions were included, and Dutch GP guidelines recommend that suspected YOD cases should be referred to memory clinics for diagnostic work-up [20, 41]. Our data supports this, showing that only 0.5% of diagnoses in the Rotterdam area and 2.2% in the Eindhoven-De Kempen area were made by GPs. This finding is consistent with another Dutch study that reported that 96% of YOD cases were referred to a neurologist or geriatrician [27]. Combining our incidence data, with an average disease duration of 6.9 to 9.3 years [17, 28, 51], we estimate that between 14,655 and 20,340 individuals were living with YOD in the Netherlands in 2021. These estimates are consistent with previous estimated national figures, such as those reported by Hendriks et al. (14,000—17,000), based on international estimates. An additional consideration for interpreting YOD burden estimates is the age range included. While several studies restrict prevalence or incidence estimates to individuals aged 45–64 years (e.g. [39, 50]), our findings demonstrate that YOD also occurs in younger age groups. Excluding these younger individuals may therefore underestimate the overall burden of YOD and overlook the specific needs associated with a very young onset.

When examining dementia subtypes, FTD was the second most prevalent subtype in the Eindhoven-De Kempen area, and the third most prevalent subtype in the Rotterdam area, without significant differences. Comparisons with international studies should be interpreted with caution though, as definitions and inclusion criteria for FTD vary. In the present study, FTD diagnoses were based on clinical diagnoses reported by healthcare professionals and included bvFTD and PPA, without confirmation of underlying pathology, limiting comparability with studies incorporating biomarker or neuropathological diagnoses of frontotemporal lobar degeneration (FTLD; e.g. [9]). Some studies include corticobasal syndrome and progressive supranuclear palsy within the FTD spectrum (e.g. [9, 11]), whereas others analyze subtypes separately (e.g. [3, 23, 39, 50, 66]). These methodological differences should be considered when interpreting reported FTD prevalence and incidence estimates.

When comparing outcomes between the two regions, we found significant higher prevalence and incidence of VaD in the urban Rotterdam region compared to the more rural Eindhoven-De Kempen region. One possible explanation is the difference in SES, with individuals in the Rotterdam area scoring lower (−0.25, below the national mean) than those in Eindhoven-De Kempen (0.04, slightly above the national mean), as lower SES is related to increased VaD risk factors such as smoking, alcohol consumption and cardiovascular disease [42, 44, 52]. While SES differences might play a role, other factors, such as urban–rural differences in diagnostic work-up, could also contribute [53].

Another notable difference between the two regions was the diagnostic delay (2.2 years in the Rotterdam area vs 1.3 years in the Eindhoven-De Kempen area). Interestingly, this pattern contrasts with findings from previous studies reporting longer diagnostic delays in rural areas (e.g. [2, 21, 48]). The explanation of this difference can be that in the Netherlands rurality does not imply limited access to care, as the country is densely populated and specialized care services are generally available within short travel distances, often with shorter waiting times for care compared to urban regions [63]. The difference in regional diagnostic delay might therefore be explained by variations in access to care, waiting times, diagnostic work-up and diagnosing specialists [53, 57, 63], or age distribution, as a younger onset has been associated with longer diagnostic delays [9, 13]. The overall delay observed in our study was shorter than previous Dutch estimates of 4.4 years [62] and a recent international systematic review reporting 4.1 years between symptom onset and diagnosis [30]. This difference may reflect our use of the first GP consultation as the starting point, rather than symptom onset, which previous research suggested occurs on average 2.3 years before the first GP consultation [13]. Finally, in the present study, duplicate cases—where the same individual with YOD was registered by multiple professionals—showed some inconsistencies in reported dates of first GP consultation, indicating limited reliability of this measure. This also applies to estimates of first symptoms, as these are typically based on retrospective recall and may therefore be prone to some inaccuracy [13, 30].

Our study revealed important demographic and care-related findings alongside our primary prevalence and incidence results. In both catchment areas, a substantial proportion of individuals with YOD had a migration background (30.8% in the Rotterdam area and 12.5% in the Eindhoven-De Kempen area), reflecting the broader demographic context of these regions, with a larger migrant population in the urban Rotterdam area. Previous literature suggests a higher prevalence of dementia among people with a migration background in the Netherlands [45], yet inequalities in dementia recognition and access to care among migrant populations [56, 65]. Based on this literature, underrepresentation of individuals with a migration background might be expected in care-based case-finding studies. Based on our data however, we cannot infer about prevalence and incidence of YOD, and under or overrepresentation of specifically people with a migration background. Comparing the proportion of people with YOD and a migration background to the general population is inappropriate since the age distribution of the population of people with a migration background likely differs from the general population due to country specific migration patterns. Detailed age-specific population data for all different countries of origin were not available for the catchment regions, and the presence of missing data on ethnicity further complicates such comparisons. Our findings do show that a considerable and diverse group of individuals with a migration background reach YOD care services, underscoring the need for care that is both age-appropriate and culture sensitive.

Another important finding is that most individuals with YOD lived at home, either with family or alone (76.4% in the Rotterdam area and 85.4% in the Eindhoven-De Kempen area), while the minority resided in nursing homes, senior housing, or other institutional settings (23.6% and 14.6%, respectively). This underscores the need to support informal caregivers, who frequently experience physical and psychological strain and report a lower quality of life [40], but also highlights the vulnerability of individuals with YOD living alone, who are at increased risk of financial hardship [4], poorer health outcomes, and reduced quality of life [10]. Although evidence specifically linking living alone to crisis admissions in YOD is lacking, the absence of daily support may place individuals at increased risk of unmet needs or crisis situations. Tailored support, such as regular check-ins or specialized case management, may help them maintain independence, and well-being.

A key strength of our study is the use of an active case-finding approach, validated with manually verified GP data. By including both an urban and a more rural region, we aimed to ensure that our findings are representative of the broader Dutch population, while also investigating regional differences. However, several limitations should be acknowledged. First, we relied on clinical diagnoses registered by healthcare professionals and did not have access to the original clinical or pathophysiological information used to establish a diagnosis. YOD diagnoses in the Netherlands are established by memory clinics, and memory clinic specialists work in compliance with the Dutch multidisciplinary guideline for dementia [14, 20]. However, variation in diagnostic work-up between memory clinics may exist, with for example some that make use of AD biomarkers and others that do not. This can affect the reliability of the registered diagnoses. Second, our approach depended on the active involvement of healthcare professionals. Although we made every effort to contact all relevant healthcare professionals working with people with YOD in the catchment areas, it is likely that some were not reached, and not all contacted healthcare professionals were willing to participate. Third, because our estimates are based on registrations by care professionals, and thus consider care use, certain populations with YOD who make less use of, or have reduced access to dementia care may be underrepresented, as has been shown previously in the Netherlands [59, 65]. Fourth, our measurements covered only one year, potentially missing individuals diagnosed earlier who did not yet require care, possibly leading to an underestimation of prevalence, although our inclusion of waiting-list registrations may have reduced this risk slightly. Finally, data collection coincided with the onset of the COVID-19 pandemic, which probably reduced healthcare use [58, 64] and the number of recorded YOD cases. This effect likely had the greatest impact on incidence, as fewer people sought GP care during the pandemic [58], resulting in a reduction in new diagnoses [64].

In conclusion, we estimate that between 14,655 and 20,340 individuals were living with YOD in the Netherlands in 2021. This estimate is likely an underestimation, given the fact that data collection took place during the COVID‑19 pandemic. Of these, approximately 75 −85% were living at home, either alone or with family members. Although overall YOD prevalence and incidence were similar across the two regions, we found a notably higher prevalence of VaD in the urban region, underscoring the importance of region-specific cardiovascular prevention strategies, especially in areas with a lower SES. Our findings further show that a considerable and diverse group of individuals with a migration background accesses YOD care services, emphasizing the need for care pathways that are not only age-appropriate, but also person-centered and culture sensitive.

Finally, our results demonstrate that methodological choices can greatly affect prevalence and incidence estimates, which should always be considered when interpreting epidemiological data. We advocate for prospective registration of YOD to achieve more accurate prevalence estimates and to facilitate better-informed healthcare planning at the regional level. The Dutch Young Onset Dementia-INCLUDED (YOD-INCLUDED) research consortium supports this goal through initiatives that include tailoring diagnostic and post-diagnostic care to the needs of people with YOD, and prospective registration through the YOD-cohort (https://www.yod-included.nl). Such efforts offer a promising step towards more inclusive and responsive YOD care.

## Supplementary Information

Supplementary Material 1.

## Data Availability

The datasets generated and analysed during the current study contain potentially identifiable information and cannot be shared publicly for reasons of participant privacy. Pseudonymised subsets of the data may be made available from the corresponding author upon reasonable request.

## Abbreviations

AD: Alzheimer’s disease
CBS: Central Bureau for Statistics
CI: Confidence interval
DPIA: Data Protection Impact Assessment
FTD: Frontotemporal dementia
GP: General practitioner
ICPC: International Classification of Primary Care
IPCI: Integrated Primary Care Information database
PRECODE: Prevalence Recognition and Care Pathways in Young Onset Dementia
RPCD: Rijnmond Primary Care Database
SD: Standard deviation
SES: Socioeconomic status
VaD: Vascular dementia
YOD: Young-onset dementia

## References

[1] Ahmad OB, Boschi-Pinto C, Lopez AD, Murray CJ, Lozano R, Inoue M. Age standardization of rates: a new WHO standard. Geneva: World Health Organization. 2001;9(10):1–14.

[2] Arsenault-Lapierre G, Bui TX, Le Berre M, Bergman H, Vedel I. Rural and urban differences in quality of dementia care of persons with dementia and caregivers across all domains: a systematic review. BMC Health Serv Res. 2023;23(1):102. 10.1186/s12913-023-09100-8. 36721162 PMC9887943

[3] Awata S, Edahiro A, Arai T, Ikeda M, Ikeuchi T, Kawakatsu S, et al. Prevalence and subtype distribution of early-onset dementia in Japan. Psychogeriatrics. 2020;20(6):817–23. 10.1111/psyg.12596. 32815229

[4] Bagnasco G, Bakx P, Licher S, van Exel J, Wouterse B. Earnings losses in young-onset dementia: Population-based study with admin data. Alzheimers Dement. 2025;21(2):e14588. 10.1002/alz.14588. 39988635 PMC11847646

[5] Barnes J, Dickerson BC, Frost C, Jiskoot LC, Wolk D, van der Flier WM. Alzheimer’s disease first symptoms are age dependent: Evidence from the NACC dataset. Alzheimers Dement. 2015;11(11):1349–57. 10.1016/j.jalz.2014.12.007. 25916562 PMC4619185

[6] Berenschot. Onderzoek naar het zorgaanbod voor mensen met dementie op jonge leeftijd [Study on care services for people with young-onset dementia]. Ministry of Health, Welfare and Sport. 2022. https://open.overheid.nl/documenten/ronl-17d7a46415429d076f72219707e10330f5b08c25/pdf.

[7] Centraal Bureau voor de Statistiek (CBS). StatLine - Bevolking; migratieachtergrond, generatie, lft, regio, 1 jan; 2010–2022 Accessed 11 January 2019 and 8 March 2023

[8] Chiari A, Vinceti G, Adani G, Tondelli M, Galli C, Fiondella L, et al. Epidemiology of early onset dementia and its clinical presentations in the province of Modena. Italy Alzheimers Dement. 2021;17(1):81–8. 10.1002/alz.12177. 32914938

[9] Chiari A, Tondelli M, Galli C, Carbone C, Fiondella L, Salemme S, et al. How long does it take to diagnose young-onset dementia? A comparison with late-onset dementia. Neurol Sci. 2022;43(8):4729–34. 35435594 10.1007/s10072-022-06056-1

[10] Clare L, Gamble LD, Martyr A, Henderson C, Knapp M, Matthews FE, team Is. Living Alone With Mild-to-Moderate Dementia Over a Two-Year Period: Longitudinal Findings From the IDEAL Cohort. Am J Geriatr Psychiatry. 2024;32(11):1309–1321. 10.1016/j.jagp.2024.05.012. 38897833

[11] Coyle-Gilchrist IT, Dick KM, Patterson K, Vázquez Rodríquez P, Wehmann E, Wilcox A, et al. Prevalence, characteristics, and survival of frontotemporal lobar degeneration syndromes. Neurology. 2016;86(18):1736–43. 10.1212/WNL.0000000000002638. 27037234 PMC4854589

[12] de Ridder MAJ, de Wilde M, de Ben C, Leyba AR, Mosseveld BMT, Verhamme KMC, et al. Data Resource Profile: The Integrated Primary Care Information (IPCI) database, The Netherlands. Int J Epidemiol. 2022;51(6):e314–23. 10.1093/ije/dyac026. 35182144 PMC9749682

[13] Draper B, Cations M, White F, Trollor J, Loy C, Brodaty H, et al. Time to diagnosis in young-onset dementia and its determinants: the INSPIRED study. Int J Geriatr Psychiatry. 2016;31(11):1217–24. 26807846 10.1002/gps.4430

[14] Federatie Medisch Specialisten. Diagnostische criteria [Diagnostic criteria]. In Dementie en lichte cognitieve stoornissen [Dementia and mild cognitive impairment]. 2014. https://richtlijnendatabase.nl/richtlijn/dementie_en_lichte_cognitieve_stoornissen_mild_cognitive_impairment_mci/diagnostiek_cognitieve_stoornissen_en_dementie/diagnostische_criteria.html.

[15] Fonseka L, Wang D, Ryan B, Cheung G, Ma’u E. Incidence of Young Onset Dementia in Waikato, New Zealand: A Population-Based Study. J Alzheimers Dis. 2022;90(3):1321–7. 10.3233/JAD-220802. 36245382

[16] Garre-Olmo J, Genís Batlle D, del Mar Fernández M, Marquez Daniel F, de Eugenio Huélamo R, Casadevall T, Turbau Recio J, Turon Estrada A, López-Pousa S, Registry of Dementia of Girona Study Group. Incidence and subtypes of early-onset dementia in a geographically defined general population. Neurology. 2010;75(14):1249-1255. 10.1212/WNL.0b013e3181f5d4c4 20810999

[17] Gerritsen AAJ, Bakker C, Verhey FRJ, Pijnenburg YAL, Millenaar JK, de Vugt ME, et al. Survival and life-expectancy in a young-onset dementia cohort with six years of follow-up: the NeedYD-study. Int Psychogeriatr. 2019;31(12):1781–9. 10.1017/S1041610219000152. 30915930

[18] Giebel C, Readman MR, Godfrey A, Gray A, Carton J, Polden M. Geographical inequalities in dementia diagnosis and care: A systematic review. Int Psychogeriatr. 2025;37(3):100051. 10.1016/j.inpsyc.2025.100051. 39986949 PMC12149024

[19] Gorno-Tempini ML, Hillis AE, Weintraub S, Kertesz A, Mendez M, Cappa SF, et al. Classification of primary progressive aphasia and its variants. Neurology. 2011;76(11):1006–14. 10.1212/WNL.0b013e31821103e6. 21325651 PMC3059138

[20] Gruters AAA, Ramakers I, Kessels RPC, Bouwman FH, Olde Rikkert MGM, Blom MM, et al. Development of memory clinics in the Netherlands over the last 20 years. Int J Geriatr Psychiatry. 2019;34(8):1267–74. 10.1002/gps.5132. 31034652 PMC6767517

[21] Gulline H, Carmody S, Yates M, Bevins A, Brodtmann A, Loi SM, et al. Equity of access in rural and metropolitan dementia diagnosis, management, and care experiences: an exploratory qualitative study. Int J Equity Health. 2025;24(1):74. 10.1186/s12939-025-02434-1. 40091013 PMC11912628

[22] Gumus M, Multani N, Mack ML, Tartaglia MC, Alzheimer's Disease Neuroimaging I. Progression of neuropsychiatric symptoms in young-onset versus late-onset Alzheimer's disease. Geroscience. 2021;43(1):213–223. 10.1007/s11357-020-00304-y. PMC8050126 33420706

[23] Harvey RJ, Skelton-Robinson M, Rossor MN. The prevalence and causes of dementia in people under the age of 65 years. J Neurol Neurosurg Psychiatry. 2003;74(9):1206–9. 10.1136/jnnp.74.9.1206. 12933919 PMC1738690

[24] Hendriks S, Peetoom K, Bakker C, van der Flier WM, Papma JM, Koopmans R, Verhey FRJ, de Vugt M, Köhler S, Young-Onset Dementia Epidemiology Study Group. Global prevalence of young-onset dementia: a systematic review and meta-analysis. JAMA Neurology. 2021;78(9):1080-1090. 10.1001/jamaneurol.2021.2161. PMC8290331 34279544

[25] Hendriks S, Peetoom K, Tange H, van Bokhoven MA, van der Flier WM, Bakker C, et al. Pre-Diagnostic Symptoms of Young-Onset Dementia in the General Practice up to Five Years Before Diagnosis. J Alzheimers Dis. 2022;88(1):229–39. 35570494 10.3233/JAD-220215 PMC9277692

[26] Hendriks S, Peetoom, K, Bakker C, Koopmans R, van der Flier WM, Papma JM, Verhey F, Young-Onset Dementia Epidemiology Study Group, de Vugt M, Köhler S. Global incidence of young-onset dementia: a systematic review and meta-analysis. Alzheimers Dement. 2023a;19(3):831-843. 10.1002/alz.12695. 35715891

[27] Hendriks S, Peetoom K, Tange H, Papma JM, van der Flier WM, Koopmans R, et al. Diagnosis and care use for people with young-onset dementia in primary care in the Netherlands. J Alzheimers Dis. 2023;91(2):653–62. 36502322 10.3233/JAD-220713 PMC9912727

[28] Joling KJ, Janssen O, Francke AL, Verheij RA, Lissenberg-Witte BI, Visser PJ, et al. Time from diagnosis to institutionalization and death in people with dementia. Alzheimers Dement. 2020;16(4):662–71. 10.1002/alz.12063. 32072728 PMC7984226

[29] Krüger J, Aaltonen M, Aho K, Heikkinen S, Kivisild A, Lehtonen A, et al. Incidence and Prevalence of Early-Onset Dementia in Finland. Neurology. 2024;103(4):e209654. 10.1212/WNL.0000000000209654. 39047214 PMC11314947

[30] Kusoro O, Roche M, Del-Pino-Casado R, Leung P, Orgeta V. Time to Diagnosis in Dementia: A Systematic Review With Meta-Analysis. Int J Geriatr Psychiatry. 2025;40(7):e70129. 40716451 10.1002/gps.70129 PMC12300619

[31] Kvello-Alme M, Brathen G, White LR, Sando SB. The Prevalence and Subtypes of Young Onset Dementia in Central Norway: A Population-Based Study. J Alzheimers Dis. 2019;69(2):479–87. 10.3233/JAD-181223. 31006688 PMC6598022

[32] Kvello-Alme M, Brathen G, White LR, Sando SB. Incidence of Young Onset Dementia in Central Norway: A Population-Based Study. J Alzheimers Dis. 2020;75(3):697–704. 10.3233/JAD-191307. 32310170 PMC7369096

[33] Logroscino G, Piccininni M, Graff C, Hardiman O, Ludolph AC, Moreno F, Otto M, Remes AM, Rowe JB, Seelaar H, Solje E, Stefanova E, Traykov L, Jelic V, Rydell MT, Pender N, Anderl-Straub S, Barandiaran M, Gabilondo A, Krüger J, FRONTIERS group. Incidence of Syndromes Associated With Frontotemporal Lobar Degeneration in 9 European Countries. JAMA Neurology. 2023;80(3):279–286. 10.1001/jamaneurol.2022.5128. PMC9887528 36716024

[34] Loi SM, Cations M, Velakoulis D. Young-onset dementia diagnosis, management and care: a narrative review. Med J Aust. 2023;218(4):182–9. 10.5694/mja2.51849. 36807325 PMC10952480

[35] McKeith IG, Boeve BF, Dickson DW, Halliday G, Taylor JP, Weintraub D, et al. Diagnosis and management of dementia with Lewy bodies: Fourth consensus report of the DLB Consortium. Neurology. 2017;89(1):88–100. 10.1212/WNL.0000000000004058. 28592453 PMC5496518

[36] McKhann GM, Knopman DS, Chertkow H, Hyman BT, Jack CR Jr, Kawas CH, et al. The diagnosis of dementia due to Alzheimer’s disease: recommendations from the National Institute on Aging-Alzheimer’s Association workgroups on diagnostic guidelines for Alzheimer’s disease. Alzheimer’s Dementia. 2011;7(3):263–9. 10.1016/j.jalz.2011.03.005. 21514250 PMC3312024

[37] McMurtray A, Clark DG, Christine D, Mendez MF. Early-onset dementia: frequency and causes compared to late-onset dementia. Dement Geriatr Cogn Disord. 2006;21(2):59–64. 10.1159/000089546. 16276111

[38] Mendez MF. The accurate diagnosis of early onset dementia. Int J Psychiatry Med. 2006;36(4):401–12. 10.2190/Q6J4-R143-P630-KW41. 17407994

[39] Mercy L, Hodges JR, Dawson K, Barker RA, Brayne C. Incidence of early-onset dementias in Cambridgeshire. United Kingdom Neurology. 2008;71(19):1496–9. 10.1212/01.wnl.0000334277.16896.fa. 18981371

[40] Millenaar JK, Bakker C, Koopmans RT, Verhey FR, Kurz A, de Vugt ME. The care needs and experiences with the use of services of people with young-onset dementia and their caregivers: a systematic review. Int J Geriatr Psychiatry. 2016;31(12):1261–76. 10.1002/gps.4502. 27271788

[41] Nederlands Huisartsengenootschap. Dementie. NHG. 2020. https://richtlijnen.nhg.org/standaarden/dementie.

[42] O’Brien JT, Thomas A. Vascular dementia. Lancet. 2015;386(10004):1698–706. 10.1016/S0140-6736(15)00463-8. 26595643

[43] Pace M, Lanzieri G, Glickman M, Zupanič T. Revision of the European Standard Population: report of Eurostat's task force. Publications Office of the European Union. 2013.

[44] Pampel FC, Krueger PM, Denney JT. Socioeconomic Disparities in Health Behaviors. Annu Rev Sociol. 2010;36:349–70. 10.1146/annurev.soc.012809.102529. 21909182 PMC3169799

[45] Parlevliet JL, Uysal-Bozkir O, Goudsmit M, van Campen JP, Kok RM, Ter Riet G, et al. Prevalence of mild cognitive impairment and dementia in older non-western immigrants in the Netherlands: a cross-sectional study. Int J Geriatr Psychiatry. 2016;31(9):1040–9. 10.1002/gps.4417. 26799690

[46] Podhorna J, Winter N, Zoebelein H, Perkins T, Walda S. Alzheimer’s Diagnosis: Real-World Physician Behavior Across Countries. Adv Ther. 2020;37(2):883–93. 10.1007/s12325-019-01212-0. 31933051 PMC7004426

[47] R Core Team. R: A language and environment for statistical computing (Version 4.3.0) [Software]. R Foundation for Statistical Computing. 2023. https://www.R-project.org/.

[48] Rahman M, White EM, Mills C, Thomas KS, Jutkowitz E. Rural-urban differences in diagnostic incidence and prevalence of Alzheimer’s disease and related dementias. Alzheimer’s Dementia. 2021;17(7):1213–30. 10.1002/alz.12285. 33663019 PMC8277695

[49] Rascovsky K, Hodges JR, Knopman D, Mendez MF, Kramer JH, Neuhaus J, et al. Sensitivity of revised diagnostic criteria for the behavioural variant of frontotemporal dementia. Brain. 2011;134(Pt 9):2456–77. 10.1093/brain/awr179. 21810890 PMC3170532

[50] Ratnavalli E, Brayne C, Dawson K, Hodges JR. The prevalence of frontotemporal dementia. Neurology. 2002;58(11):1615–21. 10.1212/wnl.58.11.1615. 12058088

[51] Rhodius-Meester HFM, Tijms BM, Lemstra AW, Prins ND, Pijnenburg YAL, Bouwman F, Scheltens P, Flier WMvd. Survival in memory clinic cohort is short, even in young-onset dementia. J Neurol Neurosurg Psychiatry. 2019;90:726–728. 10.1136/jnnp-2018-318820. PMC6581097 30045942

[52] Rizzi L, Rosset I, Roriz-Cruz M. Global epidemiology of dementia: Alzheimer’s and vascular types. Biomed Res Int. 2014;2014:908915. 10.1155/2014/908915. 25089278 PMC4095986

[53] Roheger M, Eriksdotter M, Westling K, Kalbe E, Garcia-Ptacek S. Basic diagnostic work-up is more complete in rural than in urban areas for patients with dementia: results of a Swedish dementia registry study. J Alzheimers Dis. 2019;69(2):455–62. 10.3233/JAD-190017. 30988239 PMC6597969

[54] Ryan B, To E, Ma'u E, Chan AHY, Rivera-Rodriguez C, Curtis MA, Cullum S, Cheung G. Prevalence of young-onset dementia: nationwide analysis of routinely collected data. J Neurol Neurosurg Psychiatry. 2022. 10.1136/jnnp-2022-329126. 35995550

[55] Seath P, Macedo-Orrego LE, Velayudhan L. Clinical characteristics of early-onset versus late-onset Alzheimer’s disease: a systematic review and meta-analysis. Int Psychogeriatr. 2024;36(12):1093–109. 10.1017/S1041610223000509. 37431284

[56] Selten JP, Termorshuizen F, van Sonsbeek M, Bogers J, Schmand B. Migration and dementia: a meta-analysis of epidemiological studies in Europe. Psychol Med. 2021;51(11):1838–45. 10.1017/S0033291720000586. 32264980 PMC8381287

[57] Sharma S, Ilse C, Brickell K, Le Heron C, Woods K, O’Mara Baker A, et al. Determinants of Time to Diagnosis in Young-Onset Dementia. Am J Alzheimers Dis Other Demen. 2024;39:15333175241309524. 39689290 10.1177/15333175241309525 PMC11653468

[58] Splinter MJ, Velek P, Ikram MK, Kieboom BCT, Peeters RP, Bindels PJE, et al. Prevalence and determinants of healthcare avoidance during the COVID-19 pandemic: A population-based cross-sectional study. PLoS Med. 2021;18(11):e1003854. 10.1371/journal.pmed.1003854. 34813591 PMC8610236

[59] Strooij BT, Blom MT, van Hout HP, Maarsingh OR, Elders PJ, van Campen JP, et al. Associations between healthcare use and migration background in persons with dementia: A cohort study in the Netherlands. Aging Health Res. 2024;4(2):100191.

[60] van de Veen D, Bakker C, Peetoom K, Pijnenburg Y, Papma J, group tPs, de Vugt M, Koopmans R. Provisional consensus on the nomenclature and operational definition of dementia at a young age, a Delphi study. Int J Geriatr Psychiatry. 2022;37(3):1–11. 10.1002/gps.5691. PMC9305901 35156239

[61] van Gils AM, Rhodius-Meester HFM, Leeuwis AE, Handgraaf D, Bakker C, Peetoom K, et al. Young-onset dementia in memory clinics in the Netherlands: study design and description of PRECODE-GP. Alzheimers Dement. 2023;15(3):e12471. 10.1002/dad2.12471. PMC10441283 37609004

[62] van Vliet D, de Vugt ME, Bakker C, Pijnenburg YA, Vernooij-Dassen MJ, Koopmans RT, et al. Time to diagnosis in young-onset dementia as compared with late-onset dementia. Psychol Med. 2013;43(2):423–32. 10.1017/S0033291712001122. 22640548

[63] van Westendorp S, Hofman C, Mortier M, Appelhof B, Gerring P, Koopmans R, et al. Exploring the delivery and management of specialised post-diagnostic care and support in young-onset dementia: a cross-sectional study. Health services insights. 2025;18:11786329251388776. 10.1177/11786329251388775. 41200695 PMC12586858

[64] Velek P, Splinter MJ, Ikram MK, Ikram MA, Leening MJG, van der Lei J, et al. Changes in the Diagnosis of Stroke and Cardiovascular Conditions in Primary Care During the First 2 COVID-19 Waves in the Netherlands. Neurology. 2022;98(6):e564–72. 10.1212/WNL.0000000000013145. 34965968 PMC8829962

[65] Vissenberg R, Uysal O, Goudsmit M, van Campen J, Buurman-van Es B. Barriers in providing primary care for immigrant patients with dementia: GPs’ perspectives. BJGP Open. 2018;2(4):bjgpopen18X101610. 10.3399/bjgpopen18X101610. 30723796 PMC6348325

[66] Withall A, Draper B, Seeher K, Brodaty H. The prevalence and causes of younger onset dementia in Eastern Sydney. Australia Int Psychogeriatr. 2014;26(12):1955–65. 10.1017/S1041610214001835. 25307142

