# Supplementary material for: The prevalence and incidence of young onset dementia in the Netherlands: a prospective study in two catchment areas
Source: Alzheimers Res Ther. 2026 Jun 22;18:200. doi: 10.1186/s13195-026-02120-6 (PMC13551900; doi:10.1186/s13195-026-02120-6)
Supplement: Supplementary file 1 [file 13195_2026_2120_MOESM1_ESM.docx]

**Supplementary methods**

*Catchment areas*

The first catchment area, the Rotterdam area, included the municipalities of Rotterdam, Schiedam, Maassluis, Vlaardingen and Capelle aan den Ijssel. In 2018, 46.3% of the catchment area population had a migration background, comparable to other major Dutch cities such as Amsterdam, The Hague, and Utrecht (34.6% to 53.7%; 2019). When considering the age distribution, the Rotterdam area has a higher proportion of residents aged 20-40 years, and a lower proportion aged 50 years and older, compared to the total Dutch population (Supplementary figure 1).

The second catchment area, the Eindhoven-De Kempen area, included the municipalities of Eindhoven, Bergeijk, Best, Bladel, Cranendonck, Eersel, Geldrop-Mierlo, Heeze-Leende, Nuenen, Gerwen en Nederwetten, Oirschot, Reusel-De Mierden, Son en Breugel, Valkenswaard, Veldhoven, and Waalre. The age distribution of this region closely resembles that of the Dutch population (Supplementary figure 1).

The demographic statistics used to describe the catchment areas in the manuscript are based on 2018 population data, which were the most recent figures available when the catchment areas were defined in 2019. For the prevalence and incidence calculations, we used updated population numbers corresponding to the measurement periods: 2020 for the Rotterdam region, and 2022 for the Eindhoven-De Kempen region (CBS, 2020 and 2022). During these measurements periods, the Rotterdam region had a population of 903,619 individuals aged 30-69 years, and the Eindhoven-De Kempen region 574,241 individuals (Supplementary Table 1).

*Engagement and monitoring during data collection*

Throughout the measurement year, healthcare professionals were contacted multiple times to provide guidance and support in registering eligible individuals. Furthermore, we distributed newsletters and conducted mid-measurement evaluations, as well as end-of-year evaluations, to increase engagement and ensure that no eligible individuals were missed.

*Avoiding duplicate entries*

To prevent multiple registrations of the same individual, we created a unique identification code based on the birth month and year, the digits of the postal code, and the last three digits of the citizen service number (a unique nine-digit number assigned to all citizens in the Netherlands). This procedure was approved by the Erasmus MC Medical Ethical committee (MEC-2019-0627), and aided the identification of duplicate cases while maintaining anonymity.

*Rotterdam Primary Care Database (RPCD): data source and case selection*

The RPCD contains the pseudonymized electronic health care records of patients held by the GP, including information on first symptoms, the diagnostic trajectory, diagnoses, prescribed medication, laboratory results, referrals, and letters of medical specialists.

In the Netherlands, registration with a general practitioner (GP) is mandatory for all residents, and the GP serves as the first point of contact for health-related concerns, acting as a gatekeeper to specialized medical care. The RPCD contains information on individuals from specific municipalities, largely overlapping with our Rotterdam catchment area. In January 2020, the database comprised 111,336 individuals aged 30 to 69 years.
 To identify potential YOD cases, we searched for individuals younger than 70 years registered in the database between 2010 and 2020, with the International Classification of Primary Care (ICPC) code P70, indicating a dementia diagnosis. As case identification in the RPCD is based on recorded healthcare events, this time window was chosen to ensure complete capture of all individuals with YOD during our censor year 2020. To ensure complete case identification, we additionally searched for individuals with an ICPC code of P20—representing memory, concentration, or orientation problems—when this code co-occurred with the Dutch term for dementia in the free-text of electronic patient records. Case files from all identified cases were read by one author (HB) to confirm YOD diagnosis and compliance with the inclusion criteria. Individuals were included in the prevalence and incidence calculations only if they were still actively registered at the GP practice in the database in 2020.

**Supplementary Figures**

**Supplementary figure 1 – Age distribution of the catchment areas compared to the population of the Netherlands**

**Supplementary figure 2 – Ethnical background of the population 30-69 years in the catchment areas**

**Supplementary Tables**

| **Supplementary Table 1. Demographics of the catchment areas** | | |
| --- | --- | --- |
|  | **Rotterdam area** | **Eindhoven-De Kempen area** |
| **Region** | Urban | Rural |
| **Average Social Economic Status (SES) score*** | -0.22 | 0.05 |
| **Dutch population **** | 51% | 75% |
| **Population with a migration background **** | 49% | 25% |
|  |  |  |
| **Total population ***** | 903619 | 574241 |
| **Population aged 30-34** | 70710 (7.8%) | 38558 (6.7%) |
| **Population aged 35-39** | 61852 (6.8%) | 35581 (6.2%) |
| **Population aged 40-44** | 55450 (6.1%) | 33707 (5.9%) |
| **Population aged 45-49** | 59128 (6.5%) | 35631 (6.2%) |
| **Population aged 50-54** | 59646 (6.6%) | 40648 (7.1%) |
| **Population aged 55-59** | 58194 (6.4%) | 41225 (7.2%) |
| **Population aged 60-64** | 51430 (5.7%) | 36946 (6.4%) |
| **Population aged 65-69** | 43981 (4.9%) | 31745 (5.5%) |

***** This score was calculated by the Central Bureau for Statistics (CBS) in the Netherlands and is based on the financial well-being, educational level and recent work history of private households  ****** Percentage of the population 30-69 years
******* The numbers of the total population and different age-groups were based on the numbers of the CBS corresponding to the year in which we executed the year-prevalence measurement. This was 2020 for the Rotterdam area and 2022 for the Eindhoven-De Kempen area; The percentages shown after the specific age groups represent the percentage of the total population

| **Supplementary Table 2. 1-year crude prevalence and incidence for 5-year age bands per 100,000 individuals** | | | | | | | | | | |
| --- | --- | --- | --- | --- | --- | --- | --- | --- | --- | --- |
|  | **Rotterdam area** | | | | | **Eindhoven-De Kempen area** | | | | |
|  |  | **Prevalence** | | **Incidence** | |  | **Prevalence** | | **Incidence** | |
|  | **Population** | **N** | **Crude prevalence  (95% CI)** | **N** | **Crude incidence  (95% CI)** | **Population** | **N** | **Crude prevalence  (95% CI)** | **N** | **Crude incidence  (95% CI)** |
| **30-34** | - | - | - | - | - | - | - | - | - | - |
| **35-39** | 61852 | 4 | 6.5 (1.8-16.6) | 2 | 3.2 (0.4 -11.7) | - | - | - | - | - |
| **40-44** | 55450 | 5 | 9.0 (2.9-21.0) | 2 | 3.6 (0.4 - 13.0) | - | - | - | - | - |
| **45-49** | 59128 | 14 | 23.7 (12.9-39.7) | 2 | 3.4 (0.4 - 12.2) | 35631 | 5 | 14.0 (4.6-32.7) | 3 | 8.4 (1.7 - 24.6) |
| **50-54** | 59646 | 19 | 31.9 (19.2-49.7) | 6 | 10.1 (3.7 - 21.9) | 40648 | 10 | 24.6 (11.8-45.2) | 6 | 14.8 (5.4 - 32.1) |
| **55-59** | 58194 | 47 | 80.8 (59.3-107.4) | 15 | 25.8 (14.4 - 42.5) | 41225 | 21 | 50.9 (31.5-77.9) | 9 | 21.8 (10.0 - 41.4) |
| **60-64** | 51430 | 84 | 163.3 (130.3-202.2) | 25 | 48.6 (31.5 - 71.8) | 36946 | 60 | 162.4 (123.9-209.0) | 18 | 48.7 (28.9 - 77.0) |
| **65-69** | 43981 | 143 | 325.1 (274.1-382.9) | 47 | 106.9 (78.5 - 142.1) | 31745 | 95 | 299.3 (242.2-365.7) | 32 | 100.8 (68.9 - 142.3) |

**Supplementary table 3. Crude prevalence and incidence for 5-year age bands per 100,000 individuals in the Rijnmond Primary Care Database**

|  |  |  | **Prevalence** | | **Incidence** | |
| --- | --- | --- | --- | --- | --- | --- |
|  | **Population** | **Personyears** | **n** | **Crude prevalence (95% CI)** | **n** | **Crude incidence (95% CI)** |
| **30-34** | 14027 | 16045.3 | 0 | - | - | - |
| **35-39** | 13379 | 15734.1 | 0 | - | - | - |
| **40-44** | 12801 | 15343.2 | 0 | - | - | - |
| **45-49** | 14719 | 17342.6 | 1 | 6.8 (0.2-37.8) | - | - |
| **50-54** | 15692 | 18366.1 | 6 | 38.2 (14.0-83.2) | 2 | 10.9 (1.3-39.3) |
| **55-59** | 15503 | 18082.4 | 15 | 96.8 (54.2-159.5) | 7 | 38.7 (15.6-79.8) |
| **60-64** | 13564 | 15609.5 | 21 | 154.8 (95.9-236.6) | 7 | 44.8 (18.0-92.4) |
| **65-69** | 13944 | 13557.7 | 46 | 329.9 (241.6-439.8) | 6 | 44.3 (16.2-96.3) |

^a^ The prevalence was calculated based on the amount of patients in the database at 1-1-2020
^b^ The incidence was calculated using the amount of person-years in the database over the whole of 2020
